# The dynamics of poverty in Europe: what has changed after the great recession?

**DOI:** 10.1007/s10888-022-09527-9

**Published:** 2022-05-27

**Authors:** Chiara Mussida, Dario Sciulli

**Affiliations:** 1 Department of Economic and Social Sciences, Università Cattolica del Sacro Cuore, via Emilia Parmense, 84, 29122 Piacenza, Italy; 2 University of Chieti-Pescara, Pescara, Italy

**Keywords:** Poverty, Genuine state dependence, Europe, Dynamic probit models, Initial conditions

## Abstract

This paper provides novel evidence on the importance of the phenomenon of poverty and its heterogeneity across European countries. We analyze the determinants of poverty in Europe and their evolution over time by disentangling the role of genuine state dependence and heterogeneity.

We apply alternative dynamic probit models accounting for endogenous initial conditions and correlated random effects to the pre-Great Recession period of 2005–2008 and the post- Great Recession period of 2015–2018 using EU-SILC longitudinal datasets for a sample of European countries in order to estimate genuine state dependence and uncover the role of observable and unobservable factors in determining the risk of poverty. Our findings suggest that the degree of genuine state dependence is relevant in Europe and that it increased slightly from pre- to post-Great Recession. This suggests that measures aimed at lifting individuals out of poverty, including cash transfers, have become even more important during the Europe 2020 decade. Our analysis also reveals that Europe is characterized by an increasing scarring effect of poverty, the trend of which has been exacerbated in the post-recession period. The analysis at the country level clarifies why the evolution of genuine state dependence was heterogeneous. While a clear pattern within macro-regions does not emerge, we find an association between country-level variation in genuine state dependence and some macroeconomic indicators. Finally, our results suggest that the protective role of higher education has diminished over time, while the role of employment stability and of childcare provision during early childhood has become even more important in the post-recession period.

## Introduction

The Europe 2020 strategy for smart, sustainable, and inclusive growth was launched by the European Commission in 2010, with the aim of improving European competitiveness and productivity and to promote a sustainable social market economy. In addition to employment, research and development, climate change and energy, and education, the European agenda included targets aimed at fighting poverty and social exclusion, with the aim of appending social and territorial cohesion to economic growth. In order to achieve this objective, the European Commission set the quantitative target of lifting over 20 million people out of poverty and social exclusion by 2020.

Ten years later, although several indicators have improved the goal of substantially reducing poverty and social exclusion is far from being reached. The number of people at risk of poverty and social exclusion only decreased from 116.5 million in 2010 (23.7%) to 107.5 million in 2019 (21.4%). In some countries, including those of Southern Europe, as well as Sweden and the Netherlands, the number of people at risk of poverty and social exclusion has even increased over the last decade. In addition, the persistent at-risk-of-poverty rate has generally increased in Europe,^1^ indicating that the long-term dimension of poverty has become stronger in recent years (e.g., Giarda and Moroni 2018; Bosco and Poggi 2020).

This disappointing pattern is partly explained by the increase in unemployment and the financial distress caused by the Great Recession, which negatively affected household incomes in Europe. In addition, some countries (e.g., the southern ones) suffered particularly from the implementation of contractionary fiscal policies and labor market deregulation, which contributed to increasing the socioeconomic vulnerability of societies (Jenkins 2020).

Nevertheless, the European Union’s priority of fighting poverty is confirmed by the commitments made in the United Nations’ 2030 Agenda for Sustainable Development (2015), which includes among its 17 Sustainable Development Goals (SDGs) some objectives aimed at eradicating poverty and achieving worldwide sustainable development by 2030. Moreover, the need to adopt short- and long-term measures to combat poverty now appears even more important given the COVID-19 pandemic. In this context, understanding the forces that have driven the recent evolution of poverty outcomes is important both for a deeper knowledge of the processes steering poor economic conditions and to develop guidelines to design future anti-poverty measures. This paper contributes to the literature by analyzing how the factors driving poverty persistence in Europe have changed over time. Exploiting homogeneous information drawn from the European Union Statistics on Income and Living Conditions (EU-SILC) datasets, we analyze the poverty process in both pre- and post-Great Recession periods and identify the main determinants of poverty persistence both at the European and at the country level.

The literature (e.g., Biewen 2009; Fabrizi and Mussida 2020) has stressed that poverty is a dynamic process, and thus it is important to take a longitudinal perspective when analyzing it (Ayllón 2013). An advantage of using a dynamic approach is the possibility of isolating the contribution of genuine state dependence to poverty persistence from that due to observed and unobserved heterogeneity. The identification of genuine state dependence, which indicates how current poverty per se causes future poverty, is important both to uncover the underlying mechanisms leading to poverty and to determine the potential effectiveness of measures aimed at lifting individuals above the poverty line.

Our analysis is firstly interested in assessing whether and how genuine state dependence has changed between the pre- and post-Great Recession periods and then to understand its role in hindering the achievement of the Europe 2020 poverty targets. With this in mind, we estimate dynamic probit models, which account for endogenous initial conditions and correlated random effects. Our benchmark approach is that proposed by Wooldridge (2005), but we also adopt the Rabe-Hesketh and Skrondal (2013) technique to account for short-panel issues (Akay 2012) and implement a three-level random intercept model to account for unobserved heterogeneity both at the individual and at the country level (by following Bosco and Poggi 2020). We use data for twenty EU countries from the 2005–2008 and the 2015–2018 longitudinal sections of the EU-SILC database, which permits us to cover pre- and post-Great Recession periods and to embrace a large portion of the decade involved in the Europe 2020 strategy. Finally, poverty is measured by using the at-risk-of-poverty definition.^2^

Our empirical approach allows us to identify the contribution of genuine state dependence to poverty persistence and to reveal its evolution over time by comparing results from the two periods. We estimate our models at both the European and the country level. This enables us to characterize state dependence in Europe, as well as to uncover the existence of country-specific patterns.

Secondly, we disentangle the effects of a wide range of country-level explanatory variables and study whether and how observable variables have changed in terms of their impact on the risk of poverty across pre- and post-Great Recession periods. Identifying the protective role of certain individual and/or household characteristics (e.g., education and the presence of children) and their evolution over time can be helpful to design support policies for chronically disadvantaged individuals.

Our results show that the degree of genuine state dependence is relevant in Europe and that it has increased slightly from pre- to post-Great Recession. This suggests that policy measures implemented to help the poor, including cash transfers, have become even more important during the Europe 2020 decade because of their potential effect on the reduction of poverty persistence. Our analysis also reveals that, on average, Europe is characterized by an increasing scarring effect of poverty, the trend of which has been exacerbated in the post-Great Recession period. The analysis at the country level shows that the evolution of genuine state dependence was heterogeneous. While a clear pattern within macro-regions does not emerge, we find an association between country-level variation in genuine state dependence and some macroeconomic indicators, such as variation in GDP growth, diffusion of temporary employment, and the percentage of GDP invested in social benefits. Our analysis also indicates that the protective role of higher education has diminished over time, while the role of employment stability and childcare provision during early childhood has become even more important in the post-Great Recession period.

The paper is organized as follows. Section 2 reviews the existing literature. Section 3 presents the dataset and provides descriptive statistics. The empirical model is described in Sect. 4. Section 5 discusses the main findings, and Sect. 6 offers some concluding remarks.

## Literature review

In recent decades, poverty and economic vulnerability trends across the countries of the European Union have remained relatively flat, on average. This disappointing trend in poverty is not simply a consequence of the Great Recession, as for some countries the flattish trendline was the general rule before 2008, and in many cases also afterwards (Jenkins 2020). However, for some EU countries there was an increase in poverty, especially for certain vulnerable population categories such as young people, precarious workers, single parents, and single-earner families with children (Cappellari and Jenkins 2004; Gornick, and Jäntti 2012; Scherer and Grotti 2014; Ayllón 2015; Atkinson et al. 2017). In terms of poverty rates, the frequency and duration (persistence) of poverty spells vary systematically across countries and are somehow associated with welfare regimes (Fouarge and Layte 2005; Whelan and Maitre 2010; Barbieri and Bozzon 2016).

In the past 20 years, the literature on poverty has mainly focused on “longitudinal poverty”, offering dynamic analyses of the characteristics of households that are at risk of being permanently poor or, more generally, socially excluded. Among the single- and multi-country studies are, for example, Devicienti and Poggi (2011) and the more recent work by Fabrizi and Mussida (2020), which analyze the persistence of poverty in Italy, as well as Biewen (2009) in Germany and Ayllón (2013) in Spain, while Ayllón and Gábos (2017), Giarda and Moroni (2018), and Bosco and Poggi (2020) look at European countries more broadly.

The work by Devicienti and Poggi (2011) explored the dynamic interrelation and feedback effects between poverty and material deprivation in Italy, as well as state dependence in the two conditions, using European Community Household Panel (ECHP) data for the 1994–2001 period. They estimated a bivariate dynamic model, and their findings suggest a sizable state dependence for both poverty and material deprivation, as well as strong, positive, and statistically significant feedback effects, thus suggesting that poverty and material deprivation are mutually reinforcing.

Fabrizi and Mussida (2020) investigated the phenomena of at-risk-of-poverty, severe material deprivation, and subjective poverty in Italian households with dependent children using longitudinal (2013–2016) EU-SILC survey data. They apply correlated random effects probit models with endogenous initial conditions to assess genuine state dependence after controlling for structural household characteristics and variables related to participation in the labor market. Their findings indicate strong genuine state dependence regardless of the considered poverty measure, thus confirming the previous findings of poverty persistence in Italy (Devicienti and Poggi 2011). They also find a role of initial conditions for all measures considered.

Biewen (2009) estimated a model of state dependence in poverty that explicitly allows for possible feedback effects from past poverty to future employment and household composition in Germany using data from the German Socio-Economic Panel (GSOEP) over the 2000–2006 period. The findings suggest that poverty state dependence was sizeable and significant, as were feedback effects.

Ayllón (2013) explored the mechanisms behind poverty persistence in Spain using ECHP data for the 1994–2001 period. The author estimated first-order Markov models allowing for both initial conditions and attrition. Their findings suggest that more than 50% of the aggregate state dependence in Spain was genuine, while the remainder was explained by living with a head of household with no education, being an immigrant, or cohabiting with teenagers, among other characteristics.

Ayllón and Gábos (2017) dynamically analyzed the three indicators of poverty and social exclusion covered by the EU 2020 poverty target, namely at-risk-of-poverty, severe material deprivation, and low work intensity, focusing on state dependence and feedback effects. They used a pooled dataset from the EU-SILC panel for the years from 2004 to 2010 for eight European countries to estimate joint probit models (for each indicator) with feedback effects, accounting for initial conditions as well as the time-average of all time-varying observed variables. They found evidence of state dependence for all indicators and for all countries under investigation.

Giarda and Moroni (2018) exploited the longitudinal component of the 2009–2012 EU-SILC data for France, Italy, Spain, and the UK to estimate the degree of poverty state dependence. They estimated different specifications of a dynamic random effects probit model to disentangle the role of regional disparities within countries. Their findings suggest that there is evidence of genuine state dependence in all of those countries. In comparative terms, when not accounting for regional disparities within countries the degree of poverty persistence is highest in Italy and lowest in the UK. When regional effects are included, the degree of poverty persistence in Italy drops, suggesting that unlike other countries, in Italy regional disparities play an important role in explaining poverty state dependence.

Bosco and Poggi (2020) analyzed the relation between poverty dynamics and observable and unobservable country factors using longitudinal 2008–2011 EU-SILC data for 26 European countries. They estimated a three-level dynamic model. The three levels considered were individual, time, and country. They included micro-level determinants of poverty, country-level variables, lagged poverty, and initial conditions. They found evidence of genuine state dependence, as well as a role of the initial value of poverty. They also found important evidence of unobserved heterogeneity across individuals.

Overall, a common feature of the reviewed studies is that they find evidence of relatively high poverty persistence, especially in Southern European countries, as well as a role of unobserved heterogeneity. However, these studies did not investigate the evolution of the components of poverty over time. Given the current and renewed importance of the phenomenon of poverty and its heterogeneity across European countries, there is an important need to study its determinants and their evolution over time. We aim to help fill this gap in the literature. Inspired by the existing literature, indeed, we offer novel evidence on the determinants of poverty in Europe by disentangling the role of genuine state dependence and heterogeneity to reveal their evolution over time, comparing results from two periods: before and after the Great Recession. In addition, the comparison of two different periods enables us to gain insight into how changes in state dependence are associated with changes in macroeconomic conditions and public policies.

## Data and sample

We explore data from the longitudinal sample of the European Union Statistics on Income and Living Conditions (EU-SILC) survey for the 2005–2008 and 2015–2018 periods: before and after the Great Recession. The survey is conducted in most countries across the European Union by the relevant national institutes of statistics, using harmonized definitions and survey methodologies. The topics covered by the survey encompass living conditions, income, social exclusion, housing, work, demography, and education.

**Fig. 1:**
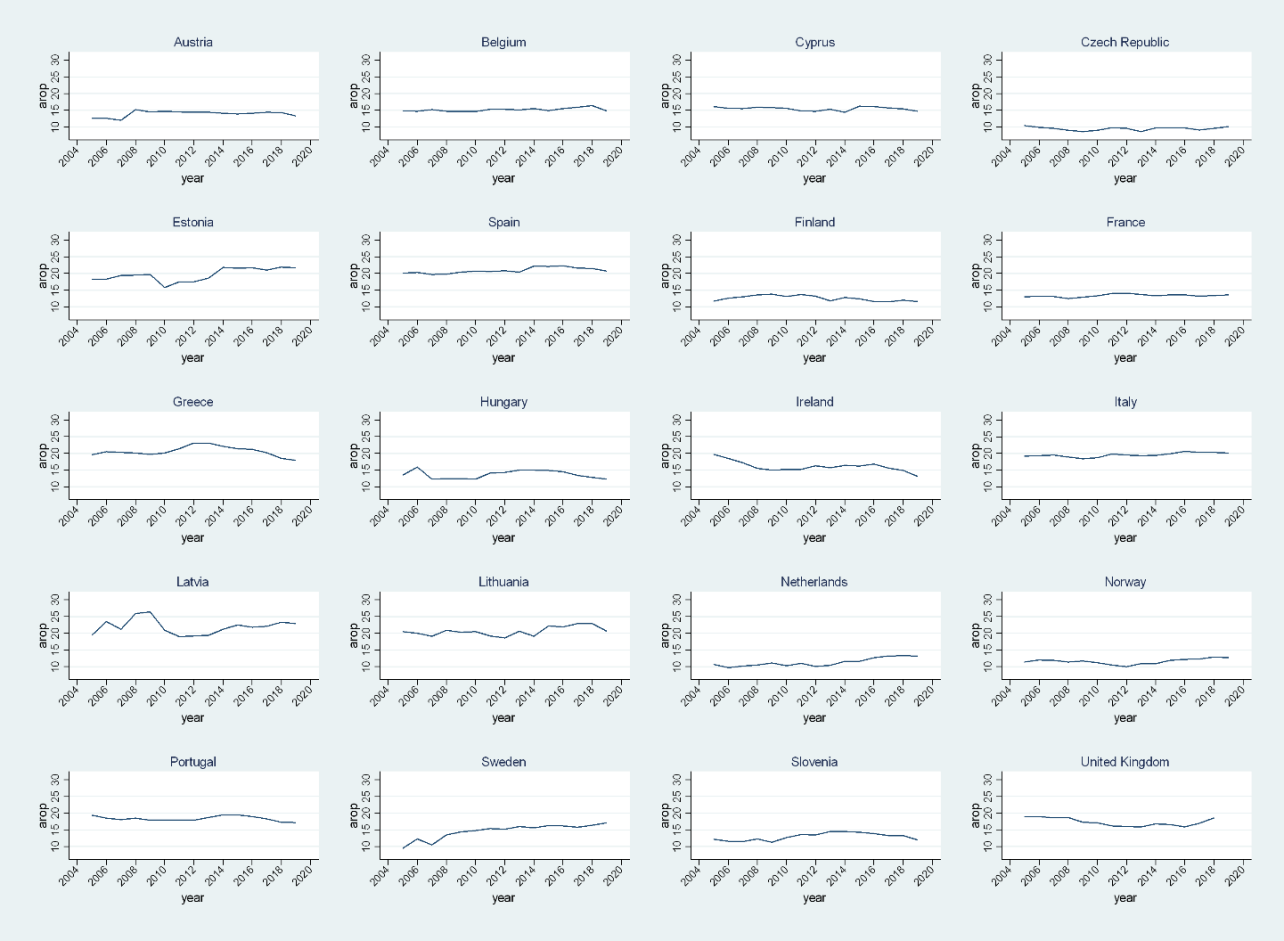
Poverty rate by country, 2005–2019. Source: Authors’ calculations from Eurostat data

We select data for twenty European countries. The EU-SILC survey includes all European countries, but we select countries for which data are available—and therefore comparable—for both periods analyzed. We focus on the phenomenon of at-risk-of-poverty, and our units of analysis are the individuals. We estimate at-risk-of-poverty by adopting different frameworks, and we compare the evolution of its main components, namely poverty genuine state dependence and (observed and unobserved) heterogeneity across a decade—from 2005 to 2008 to the more recent period of 2015–2018. At-risk-of-poverty is defined as the fraction of people living with an equivalized income below a threshold defined to be 60% of the national median. Equivalized income is defined as the total disposable household income (after taxes and social transfers) divided by an equivalized household size calculated according to the modified OECD scale.^3^ Table 1 displays the average poverty rates in the two periods analyzed for our (unbalanced) samples by country, while Fig. 1 presents the evolution of the poverty rate for the whole decade. In general, we note that the poverty rates largely differ across European countries, as does the effect of the Great Recession. From Table 1, we see that the average EU poverty rate increased by 7% points (pp.), from 15% in 2005–2008 to 22% in 2015–2018. In the first period investigated, we note that in some countries like the Netherlands and the Czech Republic the poverty rate is largely below the EU average (6.6% and 6.7%, respectively); for others it is aligned with the average, as in Ireland, France, and Hungary (poverty rate between 14% and 15%); for another group, the rate is well above the average (20.4% in Spain, 23.8% in Lithuania, and 25.9% in Greece). After the Great Recession, we again find the Netherlands and the Czech Republic with relatively lower rates, with the addition of Norway, which reduced the poverty rate from 9.2% in 2005–2008 to 7.3% in 2015–2018. There was also a reduction in the poverty rates of Hungary, France, and Finland (of 2%, on average). For Spain, Lithuania, and Greece, we still find relatively higher rates compared to the other countries explored, despite a slight reduction of the poverty rate in Lithuania and Greece (which remained above 20%, however). The change in the poverty rate due to the Great Recession by country is depicted in Fig. 1, where we clearly note that the effect was very mixed across countries.

**Table 1:** Average poverty rates. Source: Authors’ calculations from EU-SILC 2005–2008 and EU-SILC 2015–2018 data

	2005–2008		2015–2018	
	Mean	Std Dev.	Mean	Std Dev.
EU-20	0.150	0.357	0.220	0.414
Austria	0.117	0.322	0.132	0.339
Belgium	0.134	0.341	0.153	0.360
Cyprus	0.098	0.298	0.144	0.351
Czech Republic	0.067	0.250	0.087	0.281
Estonia	0.173	0.378	0.186	0.389
Spain	0.204	0.403	0.209	0.407
Finland	0.132	0.338	0.110	0.313
France	0.144	0.351	0.132	0.338
Greece	0.259	0.438	0.216	0.411
Hungary	0.145	0.352	0.116	0.320
Ireland	0.144	0.351	0.163	0.369
Italy	0.194	0.395	0.200	0.400
Latvia	0.182	0.386	0.200	0.400
Lithuania	0.238	0.426	0.210	0.407
Netherlands	0.066	0.249	0.087	0.281
Norway	0.092	0.289	0.073	0.261
Portugal	0.175	0.380	0.192	0.394
Sweden	0.100	0.300	0.114	0.318
Slovenia	0.090	0.286	0.103	0.305
United Kingdom	0.184	0.388	0.157	0.364

Table 2 reports summary statistics for the variables used in the econometric analysis in the two periods examined, as well as the relevant sample sizes. The dependent variable used in our investigation is poverty status (0, 1). We now briefly describe the covariates used, keeping in mind that due to the frameworks employed we also include lagged poverty status and initial poverty condition, as well as the average of time-varying covariates.

**Table 2:** Descriptive statistics. Source: Authors’ calculations from EU-SILC 2005–2008 and EU-SILC 2015–2018 data

	2005–2008		2015–2018	
	Mean	Std dev.	Mean	Std dev.
HH aged less than 25	0.090	0.286	0.057	0.231
HH aged 25–34	0.153	0.360	0.117	0.321
HH aged 35–44	0.244	0.429	0.213	0.409
HH aged 45–54	0.225	0.417	0.233	0.423
HH aged 55–64	0.131	0.338	0.172	0.378
HH aged more than 64	0.158	0.365	0.208	0.406
HH female	0.372	0.483	0.402	0.490
HH low educated	0.338	0.473	0.303	0.460
HH middle educated	0.449	0.497	0.408	0.491
HH highly educated	0.213	0.410	0.289	0.453
HH married	0.670	0.470	0.667	0.471
Single	0.122	0.327	0.157	0.364
Children aged 0–3	0.103	0.304	0.086	0.281
Children aged 4–15	0.368	0.482	0.328	0.469
Number of persons with disabilities	0.140	0.399	0.128	0.376
Number of elderly	0.319	0.625	0.407	0.694
Homeowner	0.907	0.291	0.795	0.404
Number of permanent employees	0.731	0.835	0.739	0.830
Number of temporary employees	0.114	0.358	0.115	0.359
Number of self-employed	0.147	0.406	0.162	0.432
Observations	237,692		370,591	

As suggested by the existing literature (see Sect. 2), our control variables can be classified primarily into individual and household characteristics. The individual characteristics refer to the characteristics of the head of household and include age (split into age ranges from less than 25 years to more than 64 years), gender, education, and marital status. The household characteristics include a control for single households, the presence of children aged from 0 to 3 years, children aged from 4 to 15, the number of disabled and elderly (aged 65 or over) in the household, home ownership, and the number of permanent employees, temporary employees, and self-employed individuals. In our models, we also control for country and time dummies.

## The econometric approach

We are interested in estimating how the determinants of poverty persistence in Europe have changed over time. With this aim, we estimate dynamic probit models with correlated random effects and endogenous initial conditions, which allows us to disentangle the contribution of genuine state dependence and (observed and unobserved) heterogeneity to poverty persistence.

The contribution of observable heterogeneity is controlled for by including in the model specification a wide range of individual and household variables. The presence of time-invariant unobserved heterogeneity can be modeled by including individual-specific random effects that are assumed to be normally distributed and independent of other covariates. However, we relax the independence assumption by adopting Mundlak’s approach (Mundlak 1978), in which the unobserved heterogeneity term is decomposed into two parts: one correlated and one uncorrelated with (time-variant) explanatory variables.

The role of state dependence is identified through the inclusion of the lagged dependent variable on the right side of the poverty equation. State dependence, however, may be consistently estimated (genuine state dependence) if the possible endogeneity between initial conditions and unobserved heterogeneity is accounted for. Heckman (1981), in fact, stressed that the state dependence parameter could be biased (spurious state dependence) in the event that the first poverty status observed in the data is affected by underlying unobservable factors conditioning the distribution of poverty at time 1. We tackle the initial conditions problem following Wooldridge (2005), who suggested an alternative conditional maximum likelihood (CML) estimator that considers the distribution conditional on the value in the initial period. An advantage of Wooldridge’s technique is that it contains Mundlak’s specification, and thus we are definitively able to estimate a correlated random effects probit model with endogenous initial conditions.

Let us define p_it_ as the individual poverty status of individual *i* = 1…*n* at time *t* = 1…*T*. According to Wooldridge’s approach, for each investigated period (2005–2008 and 2015–2018) we assume that poverty status is described by the following benchmark model:

1 $$ {p}_{it}=1\left\{\gamma {p}_{it-1}+\beta {x}_{it}+\phi {z}_{i}+{a}_{i}+{u}_{it}>0\right\}$$

where *p*_*it−1*_ is the lagged poverty status and *x*_*it*_ and *z*_*i*_ are vectors of strictly exogenous time-variant and time-invariant (respectively) individual and household characteristics. *γ* is the (genuine) state dependence parameter, and *β* and *φ* are sets of parameters to be estimated. Finally, *a*_*i*_ and *u*_*it*_ respectively represent the unobserved time-invariant individual-specific random effects and the idiosyncratic error term; we assume that these are normally distributed and that *u*_*it*_ is not serially correlated. The conditional densities of the individual-specific random effects are specified via the following auxiliary model:

2 $$ {a}_{i}={\theta }_{0}+{\theta }_{1}{p}_{i1}+{\theta }_{2}{\stackrel{-}{x}}_{i}+{\mu }_{i}$$

where *p*_*i1*_ is the initial poverty status and ${\stackrel{-}{x}}_{i}$is a set of time-averaged time-variant control variables calculated from periods 2 to T and *θ*_*k*_ are parameters to be estimated. ${\mu }_{i}$ is the residual, assumed to be normally distributed with zero mean and variance ${\sigma }_{a}^{2}$, which indicate the size of the dispersion due to the unobserved heterogeneity. In order to obtain consistent estimates, ${\mu }_{i}$ is integrated out using a numerical integration algorithm based on Gauss–Hermite quadrature at 12 points (Wooldridge 2005).

The Wooldridge approach has been questioned by Akay (2012), who stresses that the method may produce biased estimates of state dependence in case of short panels. In this regard, Rabe-Hesketh and Skrondal (2013) propose a solution that consists of including the initial period of time-varying explanatory variables as an additional regressor in the auxiliary model, with the aim of reducing the substantial finite-sample bias. Therefore, the Rabe-Hesketh and Skrondal (2013) specification reads as

3 $$ {p_{it}} = 1\{ \gamma {p_{it - 1}} + \beta {x_{it}} + \varphi {z_i} + {{a'}_i} + {u_{it}} > 0\} ,$$

where *a’*_*i*_ represents the modified unobserved time-invariant individual-specific random effects, and the auxiliary model now reads as

4 $$ {{a'}_i} = {\theta _0} + {\theta _1}{p_{i1}} + {\theta _2}{{\bar x}_i} + {\theta _3}{x_{i1}} + {\mu _i}.$$

Because the core of our study is multi-country-based, we have to consider that individuals may share common underlying unobserved characteristics with their fellow citizens, whereas they may vary among individuals living in different countries. Not accounting for country-level clustering may cause some estimation bias, including the possible underestimation of standard errors. We account for this by adopting a three-level random intercept model, which allows us to exploit the hierarchical structure of the data and to consider the possibility that for each year observations are interrelated at both the individual and country level (see Bosco and Poggi 2020 for an application). Our benchmark model is then modified as follows:

5 $$ {p_{ict}} = 1\left\{ {\gamma {p_{ict - 1}} + \beta {x_{ict}} + \varphi {z_{ic}} + {a_{ic}} + {v_c} + {u_{ict}} > 0} \right\},$$

where the subscript *c* = 1…*C* indicates the country and the additional term *v*_*c*_ indicates the random effects term at the country level, which we assume to be independently normally distributed.^4^

Wooldridge’s method and its extensions present some advantages when dealing with attrition problems that may arise when using unbalanced data. Wooldridge’s (2005) method permits attrition to vary across the initial poverty status, and in particular, individuals with different initial levels of poverty are allowed to have distinct missing-data probabilities. Therefore, the method allows easily handling attrition and reduces problems associated with the use of richer but unbalanced sets of data.^5^

Finally, because the estimated coefficients describe the sign of the relationship but are inappropriate to determine the magnitude of the impact between outcome and explanatory variables, we compute and report average marginal effects (AMEs).

## Results

We adopt different frameworks to estimate how the determinants of at-risk-of-poverty in Europe have changed over time. We compare the evolution of its main components, namely genuine poverty state dependence and (observed and unobserved) heterogeneity across a decade, that is, from 2005 to 2008 to the more recent period of 2015–2018.

Table 3 shows the estimates from the different frameworks described in detail in Sect. 4. We follow Wooldridge (2005) (columns 1 and 2) and Rabe-Hesketh and Skrondal (2013) (columns 3 and 4) and a build hierarchical model to accommodate for the hierarchical structure of the data (reported in columns 5 and 6). The first aim is to see whether there was genuine state dependence and whether this changed over the decade investigated. From the first panel of Table 3, we can observe at least three interesting results. First, there is evidence of significant genuine state dependence, confirming the previous literature on longitudinal poverty in Europe (see, for instance, Ayllón and Gábos 2017, Giarda and Moroni 2018, and Bosco and Poggi 2020). There are several mechanisms through which experiencing poverty may determine a poverty-trap effect. For example, access to social programs may disincentive individuals to adopt activities leading out of poverty in order to maintain income support. In addition, experiencing poverty may give rise to phenomena such as demoralization, loss of motivation, depreciation of human capital, and unfavorable attitudes, which may affect the chances of escaping poverty both directly and indirectly (for example, by producing detrimental effects on labor market and health outcomes; e.g., Cappellari and Jenkins 2004; Biewen 2009).

**Table 3:** Estimates of the determinants of poverty. Source: Authors’ calculations from EU-SILC 2005–2008 and EU-SILC 2015–2018 data

	Wooldridge model			Rabe-Hesketh & Skrondal model			Hierarchical model		
	2005–2008	2015–2018		2005–2008	2015–2018		2005–2008	2015–2018	
	AME	AME	Z-test	AME	AME	Z-test	AME	AME	Z-test
Poverty t-1	0.072^***^	0.075^***^	***	0.068^***^	0.072^***^	***	0.080^**^	0.087^***^	***
	(0.004)	(0.003)		(0.003)	(0.003)		(0.004)	(0.003)	
Poverty time 1	0.135^***^	0.140^***^	***	0.133^***^	0.140^***^	***	0.149^***^	0.163^***^	***
	(0.002)	(0.002)		(0.002)	(0.002)		(0.003)	(0.003)	
*Individual characteristics*									
HH aged less than 25	base-category								
HH aged 25–34	0.024^***^	0.011^***^	***	0.019^***^	0.005^*^	***	0.027^***^	0.013^***^	***
	(0.003)	(0.002)		(0.003)	(0.003)		(0.003)	(0.003)	
HH aged 35–44	0.039^***^	0.023^***^	***	0.033^***^	0.015^***^	***	0.042^***^	0.027^***^	***
	(0.003)	(0.002)		(0.003)	(0.002)		(0.003)	(0.003)	
HH aged 45–54	0.040^***^	0.032^***^		0.034^***^	0.024^***^		0.044^***^	0.038^***^	
	(0.003)	(0.002)		(0.003)	(0.002)		(0.003)	(0.003)	
HH aged 55–64	0.037^***^	0.021^***^	***	0.031^***^	0.015^***^	***	0.041^***^	0.025^***^	***
	(0.003)	(0.002)		(0.003)	(0.003)		(0.004)	(0.003)	
HH aged more than 64	0.048^***^	0.031^***^	***	0.042^***^	0.024^***^	***	0.054^***^	0.037^***^	***
	(0.004)	(0.003)		(0.004)	(0.003)		(0.004)	(0.003)	
HH female	0.013^***^	0.015^***^	*	0.014^***^	0.014^***^	***	0.015^***^	0.017^***^	**
	(0.001)	(0.001)		(0.001)	(0.001)		(0.002)	(0.001)	
HH low educated	base-category								
HH middle educated	−0.032^***^	−0.020^***^	***	−0.034^***^	−0.022^***^	***	−0.035^***^	−0.024^***^	***
	(0.002)	(0.001)		(0.002)	(0.001)		(0.002)	(0.001)	
HH highly educated	−0.080^***^	−0.057^***^	***	−0.081^***^	−0.057^***^	***	−0.088^***^	−0.066^***^	***
	(0.002)	(0.002)		(0.002)	(0.002)		(0.003)	(0.002)	
HH married	−0.042^***^	−0.031^***^		−0.042^***^	−0.027^***^	***	−0.046^***^	−0.036^***^	
	(0.004)	(0.003)		(0.004)	(0.003)		(0.004)	(0.004)	
*Household characteristics*									
Single	0.011^***^	−0.016^***^	***	0.014^***^	−0.013^***^	***	0.012^***^	−0.018^***^	***
	(0.002)	(0.002)		(0.002)	(0.002)		(0.003)	(0.002)	
Children aged 0–3	0.011^**^	0.020^***^	*	0.009^*^	0.019^***^	*	0.012^**^	0.023^***^	*
	(0.005)	(0.004)		(0.005)	(0.004)		(0.006)	(0.005)	
Children aged 4–15	0.022^***^	0.023^***^		0.022^***^	0.022^***^		0.025^***^	0.026^***^	
	(0.005)	(0.004)		(0.005)	(0.004)		(0.005)	(0.004)	
Number of persons with disabilities	−0.011^***^	0.004^*^	***	−0.011^***^	0.003^*^	***	−0.012^***^	0.004^*^	***
	(0.002)	(0.002)		(0.002)	(0.002)		(0.003)	(0.002)	
Number of elderly	−0.014^***^	−0.030^***^	***	0.014^***^	−0.030^***^	***	−0.016^***^	−0.035^***^	***
	(0.002)	(0.001)		(0.002)	(0.001)		(0.002)	(0.001)	
Homeowner	−0.033^***^	−0.035^***^	***	−0.032^***^	−0.034^***^	***	−0.036^***^	−0.042^***^	***
	(0.002)	(0.001)		(0.002)	(0.001)		(0.002)	(0.001)	
Number of permanent employed	−0.050^***^	−0.054^***^	***	−0.047^***^	−0.053^***^	***	−0.055^***^	−0.063^***^	***
	(0.003)	(0.002)		(0.002)	(0.002)		(0.003)	(0.002)	
Number of temporary employed	−0.027^***^	−0.026^***^		−0.025^***^	−0.026^***^		−0.030^***^	−0.030^***^	
	(0.003)	(0.002)		(0.003)	(0.002)		(0.003)	(0.003)	
Number of self-employed	0.001	−0.012^***^	***	0.003	−0.012^***^	***	0.001	−0.015^***^	***
	(0.002)	(0.003)		(0.002)	(0.003)		(0.002)	(0.003)	
Year dummy variables	Yes			Yes			Yes		
Country dummy variables	Yes			Yes			Yes		

Notes: Full set of control variables are included. Standard errors (in brackets). Z is the significance level of a coefficient-equality test over the two periods

*p < 0.10, **p < 0.05, ***p < 0.01

We also run chi2 (multivariate) tests on the sub-vector of all age or education coefficients which are consistent with the Z-tests. Moreover, we run a chi2 test (in the spirit of the Chow test) on the full set of coefficients which rejects the null hypothesis of parameters stability over the two periods

Second, and even more interestingly, we add novel evidence of significantly increased state dependence over time in Europe (in the decade examined) according to all of the specifications adopted.^6^^,^^7^ The increasing role of state dependence confirms the importance of designing policies aimed at addressing poverty, and especially for some target population groups, e.g., disadvantaged population categories. The importance of such policies is renewed after the Great Recession and is highly relevant nowadays as the eradication of poverty is the first SDG of the United Nations’ 2030 Agenda for Sustainable Development (2015). Given that the overall finding of an increased contribution of state dependence might be the result of mixed effects across European countries, in the next section we offer a further investigation of the issue at the country level.

Finally, we see that there is a role played by the initial poverty status. Primarily, there is significant correlation between initial conditions and unobserved heterogeneity. This stresses the importance of adopting a method dealing with initial-condition problems and confounding factors to correctly evaluate the role of state dependence. An additional interpretation provided in the literature (e.g., Ayllón 2015) suggests that jointly reading the estimates of past and initial poverty sheds light on the evolution of the trapping role of previous poverty status. If the coefficient associated with lagged poverty is smaller than that associated with initial poverty, this should be indicative that the trapping effect of previous poverty status increases over time. The opposite is true in case the coefficient associated with lagged poverty is greater than that associated with initial poverty. According to this interpretation, we would note that the trapping strength associated with previous poverty status increased significantly over time and has been reinforced in the post-Recession period.

All estimation methods we adopt confirm these findings. For instance, according to Wooldridge’s specification genuine state dependence increased significantly from 7.2 pp. in 2005–2008 to 7.5 pp.^8^ in 2015–2018, while the AME associated with the initial poverty status had a greater magnitude—almost double that of genuine state dependence—and showed a slightly greater increase from 13.5 pp. in the first period analyzed to 14 pp. in the second.

The covariates used here (described in Sect. 3) can be classified into individual and household characteristics. We see again that there are no important changes in the AMEs of the covariates across the different specifications, so we take the Wooldridge model as a reference for the interpretation of our results. The individual characteristics are the characteristics of the head of household. We find that at-risk-of-poverty is positively associated with the age of the head of household, and the magnitude of the AMEs reduce significantly over time. For instance, being a head of household aged 64 or over increased the poverty rate by 4.8 pp. in 2005–2008, and this effect reduced significantly to 3.1 pp. ten years later. A positive association with the poverty rate is also found for female heads of household (Giarda and Moroni 2018; Fabrizi and Mussida 2020), and this remained relatively unchanged over time. Notably, we find a role of education in reducing the risk of poverty, and especially for higher education. This finding is supported by previous evidence (Ayllon 2013). We also find a smaller effect (statistically significant reduction) of education after the Great Recession. This might partly be due to an issue related to sample composition, as the average level of education increased, and partly to the fact that the returns to education declined per se. Being married reduces the poverty rate as well.

In terms of household characteristics, and specifically looking at the household type, we find that the Great Recession changes the sign of the association between being a single-member household and the poverty rate. We note that while being a single-member household increases the poverty rate in 2005–2008, confirming previous work such as that of Gornick, and Jäntti (2012), Scherer and Grotti (2014), and Atkinson et al. (2017), the sign of the association reversed to negative after the Great Recession. The presence of children, instead, maintained its positive association with the poverty rate for both age categories considered, namely 0–3 years old (with a significant increase from 1.1 pp. in 2005–2008 to 2 pp. in 2015–2018) and 4 to 15 years of age (with AMEs almost unchanged: 2.2 pp. in the first period and 2.3 pp. in the second period).

Interestingly, the sign of the association between the number of disabled persons in a household and the poverty rate changed from negative before the Great Recession to positive thereafter. However, we need to add that the change in the number of disabled persons in a household, as measured by the average number of disabled persons,^9^ increases the poverty rate before the recession. The negative association may confirm a possible small role of transfer programs targeting disabled people in alleviating their poverty (Meyer and Wu 2018) since the change in the number of disabled persons remained positively associated with the poverty rate. Despite past progress in poverty reduction for the disabled, they were severely affected by the crisis (especially disabled workers), prompting calls for targeted policy interventions and transfer programs aimed at assisting disabled individuals (Brandolini and Rosolia 2019).

The presence of elderly persons in a household reduces the poverty rate before (–1.4 pp.) and especially after the Great Recession (–3 pp.). The role of the elderly as a stable source of income (especially through elderly pensions) in mitigating the risk of poverty is supported by the existing literature (see, for instance, Giarda and Moroni 2018 and Fabrizi and Mussida 2020). Being a homeowner is negatively associated with the poverty rate as well. The importance of being the owner of a home for reducing the risk of poverty is confirmed by the existing evidence (Giarda and Moroni 2018; Bosco and Poggi 2020; Fabrizi and Mussida 2020).

We control for the labor market status of household components, and we note the relevance of permanent employment (which increased slightly with the Great Recession) in reducing the poverty rate. We find a negative association for the temporarily employed as well (unchanged), while for the self-employed a negative association is found only after the Great Recession. The relevance of labor market status was also found in previous works (e.g., Barbieri and Bozzon 2016; Fabrizi and Mussida 2020).

As robustness checks, and as suggested by the existing literature (see, for instance, Jenkins 2020), due to the fact that national poverty thresholds might differ substantially in real income terms we calculate a European poverty-line threshold (EU-wide line) defined as 60% of the median equivalized household income for all European countries. We run our estimates on this EU-wide line and obtain confirmation of our main results (see Table 4).

**Table 4:** Genuine state dependence and scarring effects with EU poverty lines. Source: Authors’ calculations from EU-SILC 2005–2008 and EU-SILC 2015–2018 data.

		2005–2008	2015–2018	
		AME	AME	Z-test
Wooldridge model	Poverty t-1	0.083***	0.088***	***
		(0.004)	(0.003)	
	Poverty time 1	0.148***	0.176***	***
		(0.002)	(0.002)	
Rabe-Hesketh & Skrondal model	Poverty t-1	0.082***	0.084***	**
		(0.004)	(0.003)	
	Poverty time 1	0.146***	0.176***	***
		(0.002)	(0.002)	
Hierarchical model	Poverty t-1	0.091***	0.099***	***
		(0.004)	(0.003)	
	Poverty time 1	0.161***	0.201***	***
		(0.003)	(0.003)	

Notes: Full set of control variables are included. Standard errors (in brackets). Z is the significance level of a coefficient-equality test over the two periods.

*p < 0.10, **p < 0.05, ***p < 0.01

Finally, we also estimate our models by applying the correction for unbalancedness of data proposed by Albarran et al. (2019). On average, related estimates leave the essence of our findings unchanged. Furthermore, we estimate the models on balanced samples and do not find any changes in the main findings. For the sake of brevity, we do not report these last robustness check estimates here but they are available upon request.

### State dependence at the country level

The contribution of genuine state dependence to poverty persistence and its evolution over time is now analyzed at the country level (Table 5). Estimation results, which are obtained by applying the Wooldridge model, indicate that the magnitude of genuine state dependence varies at the country level and that its evolution does not display a unique pattern across countries in the analyzed period. In many countries, the difference in genuine state dependence is statistically significant according to the Z test (Clogg et al., 1995). In the pre-Great Recession period (i.e., 2005–2008), Greece, Portugal, and Austria displayed the highest levels of genuine state dependence (up to 0.287), while in the post-Great Recession period (i.e., 2015–2018) the highest level is confirmed for Austria (0.175) but it reduced significantly in other countries. In contrast, estimated state dependence was generally low in both periods in Belgium, Hungary, and the Netherlands (less than 0.05). Focusing on the evolution between pre- and post-Great Recession, we find that genuine state dependence decreased in eleven countries and increased in the remaining nine countries. The greatest statistically significant decrease took place in Greece, Norway, and Portugal (up to − 0.237), while the greatest statistically significant increase emerged for Spain (almost 0.1).

**Table 5:** Genuine state dependence at country level: Wooldridge model estimates. Source: Authors’ calculations from EU-SILC 2005–2008 and EU-SILC 2015–2018 data

	2005–2008	2015–2018	
	AME	AME	Z-test
Austria	0.179^***^	0.175^***^	
	(0.030)	(0.022)	
Belgium	0.031^**^	0.012	
	(0.014)	(0.017)	
Cyprus	0.026	0.123^***^	
	(0.020)	(0.016)	
Czechia	0.049^***^	0.084^***^	*
	(0.012)	(0.018)	
Estonia	0.144^***^	0.090^***^	*
	(0.008)	(0.012)	
Spain	0.040^***^	0.135^***^	***
	(0.012)	(0.017)	
Finland	0.100^***^	0.115^***^	**
	(0.025)	(0.027)	
France	0.078^***^	0.107^***^	
	(0.016)	(0.007)	
Greece	0.287^***^	0.050^***^	***
	(0.054)	(0.014)	
Hungary	0.038^***^	0.040^***^	
	(0.009)	(0.009)	
Ireland	0.002	0.062^*^	**
	(0.191)	(0.027)	
Italy	0.053^***^	0.064^***^	***
	(0.007)	(0.007)	
Lithuania	0.145^***^	0.090^***^	***
	(0.018)	(0.019)	
Latvia	0.168^***^	0.087^***^	
	(0.012)	(0.013)	
Netherlands	0.056^***^	0.030^***^	*
	(0.009)	(0.006)	
Norway	0.150^***^	0.019^*^	***
	(0.018)	(0.009)	
Portugal	0.201^***^	0.102^***^	***
	(0.018)	(0.008)	
Sweden	0.043	0.079^*^	
	(0.048)	(0.033)	
Slovenia	0.040^***^	0.027^***^	
	(0.012)	(0.007)	
United Kingdom	0.070^***^	0.038^***^	
	(0.024)	(0.013)	

Notes: Full set of control variables are included. Standard errors (in brackets). Z is the significance level of a coefficient-equality test over the two periods. *p < 0.10, **p < 0.05, ***p < 0.01

**Fig. 2:**
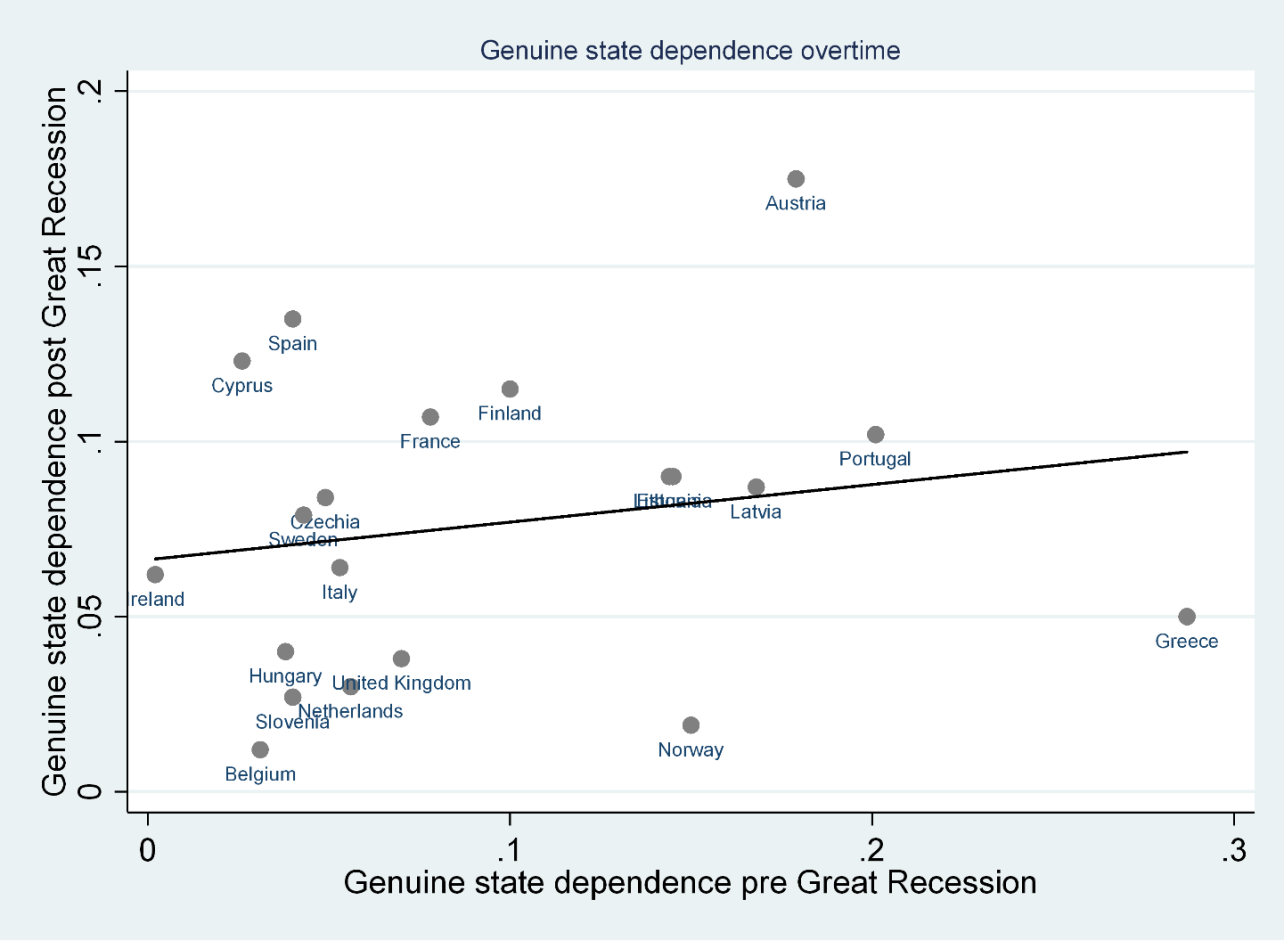
Correlation between genuine state dependence pre and post Great Recession. Source: Authors’ calculations from EU-SILC 2005–2008 and EU-SILC 2015–2018 data

Despite this composite pattern, however, we find a tendency for some countries to have strong state dependence and for others to have low state dependence (Fig. 2). Interestingly, the cross-country variability in genuine state dependence decreased in the post-Great Recession period, suggesting that its relevance for poverty persistence has become more homogeneous across countries over time. In addition, our estimates do not show a consistent pattern within macro-regions (and common welfare systems), either in terms of magnitude or evolution.

However, we focus on other potential factors that may explain the evolution of genuine state dependence in Europe in the analyzed period. We look for the existence of an association between the evolution of genuine state dependence and the evolution of some macro-indicators^10^ (i.e., social benefits, GDP growth, and share of temporary employment), which are defined as the difference between average values calculated in the 2004–2007 and 2014–2017 periods, respectively. We are aware that we cannot give a causal interpretation of these relationships, but we are confident that they provide new insights to be better explored in dedicated studies.

**Fig. 3:**
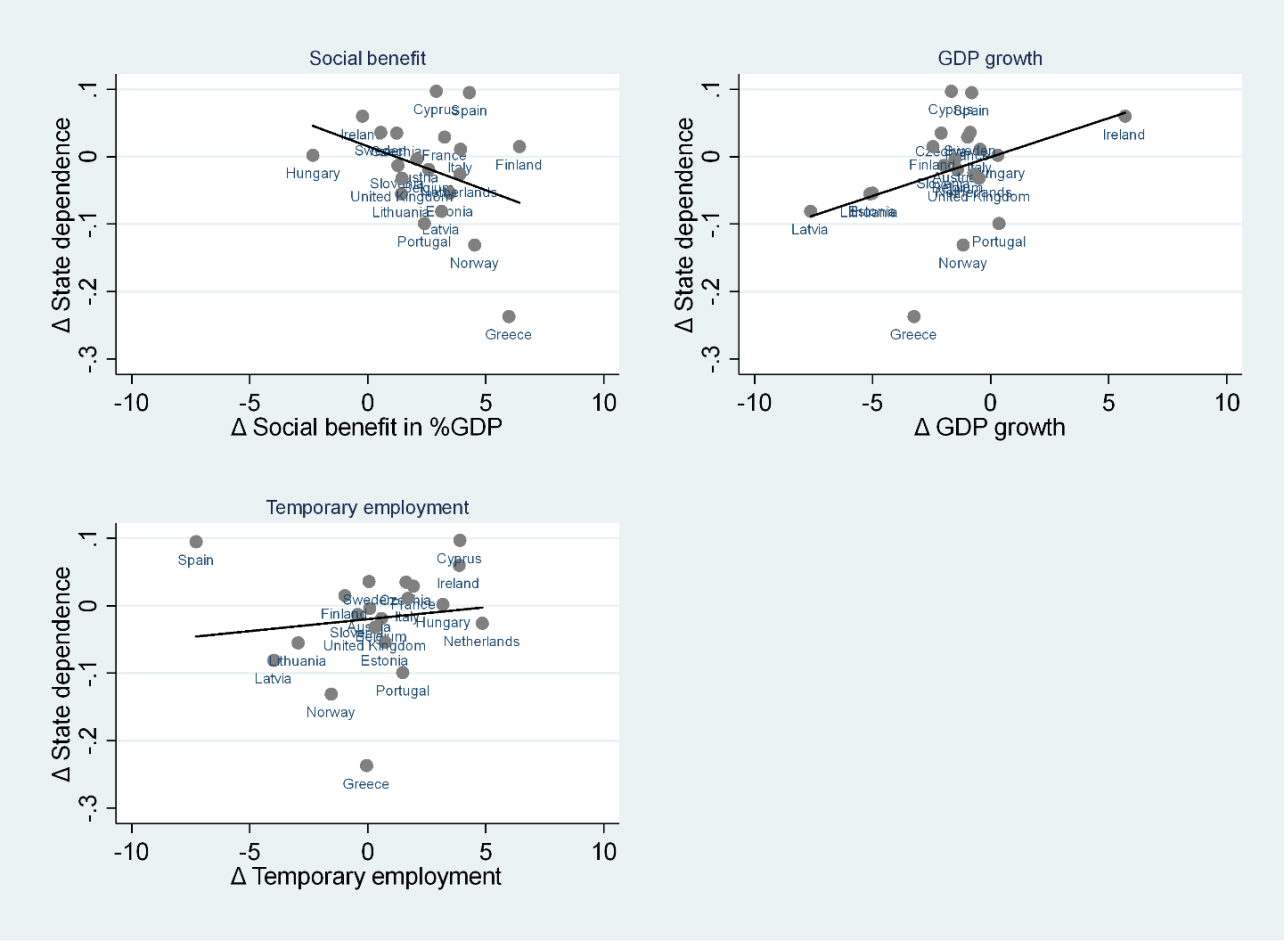
The correlation between state dependence variation and macro variables variation. Source: Authors’ calculations from EU-SILC 2005–2008 and EU-SILC 2015–2018 data

**Fig. 4:**
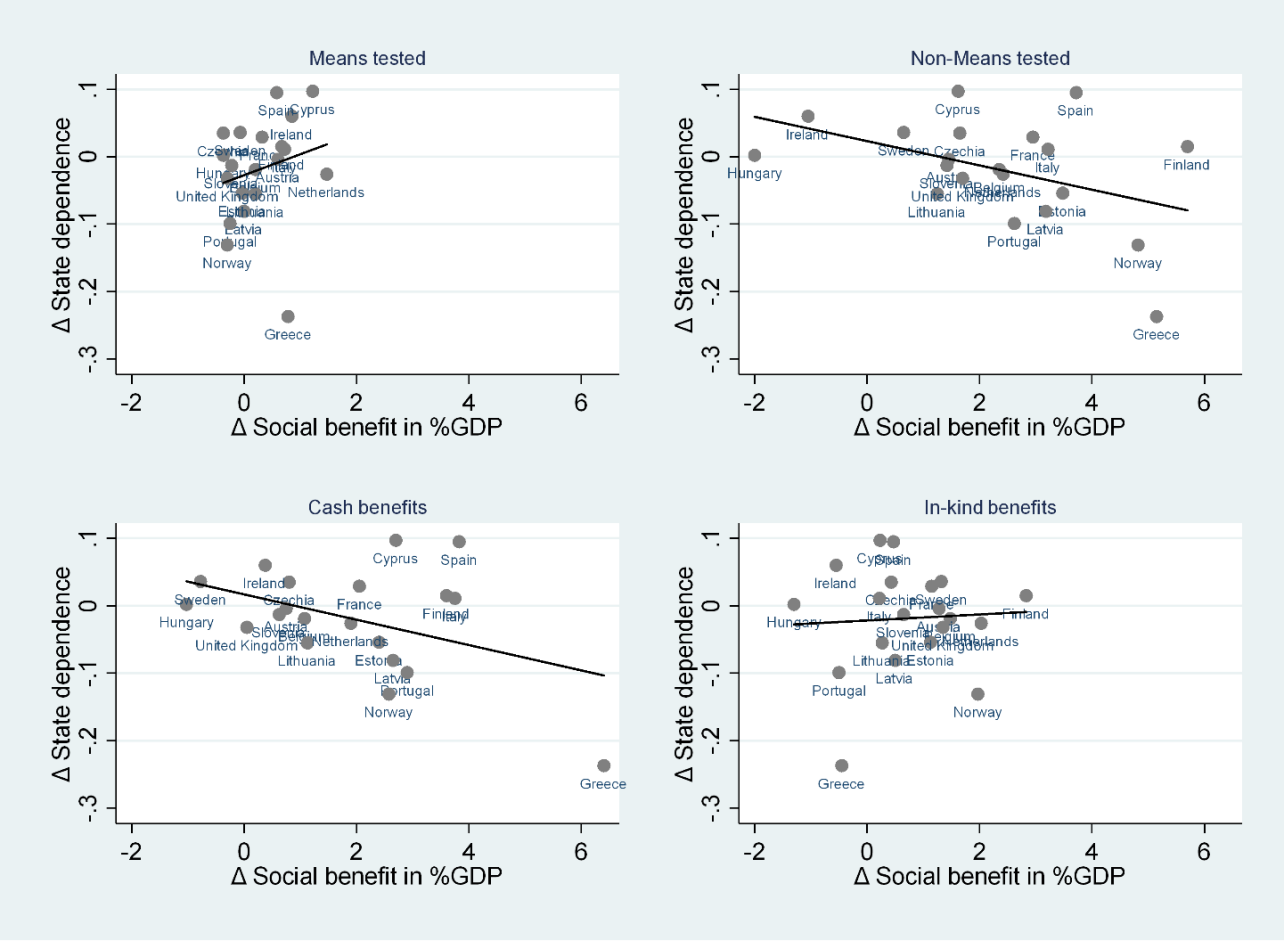
The correlation between state dependence variation and types of social benefit variation. Source: Authors’ calculations from EU-SILC 2005–2008 and EU-SILC 2015–2018 data

**Fig. 5:**
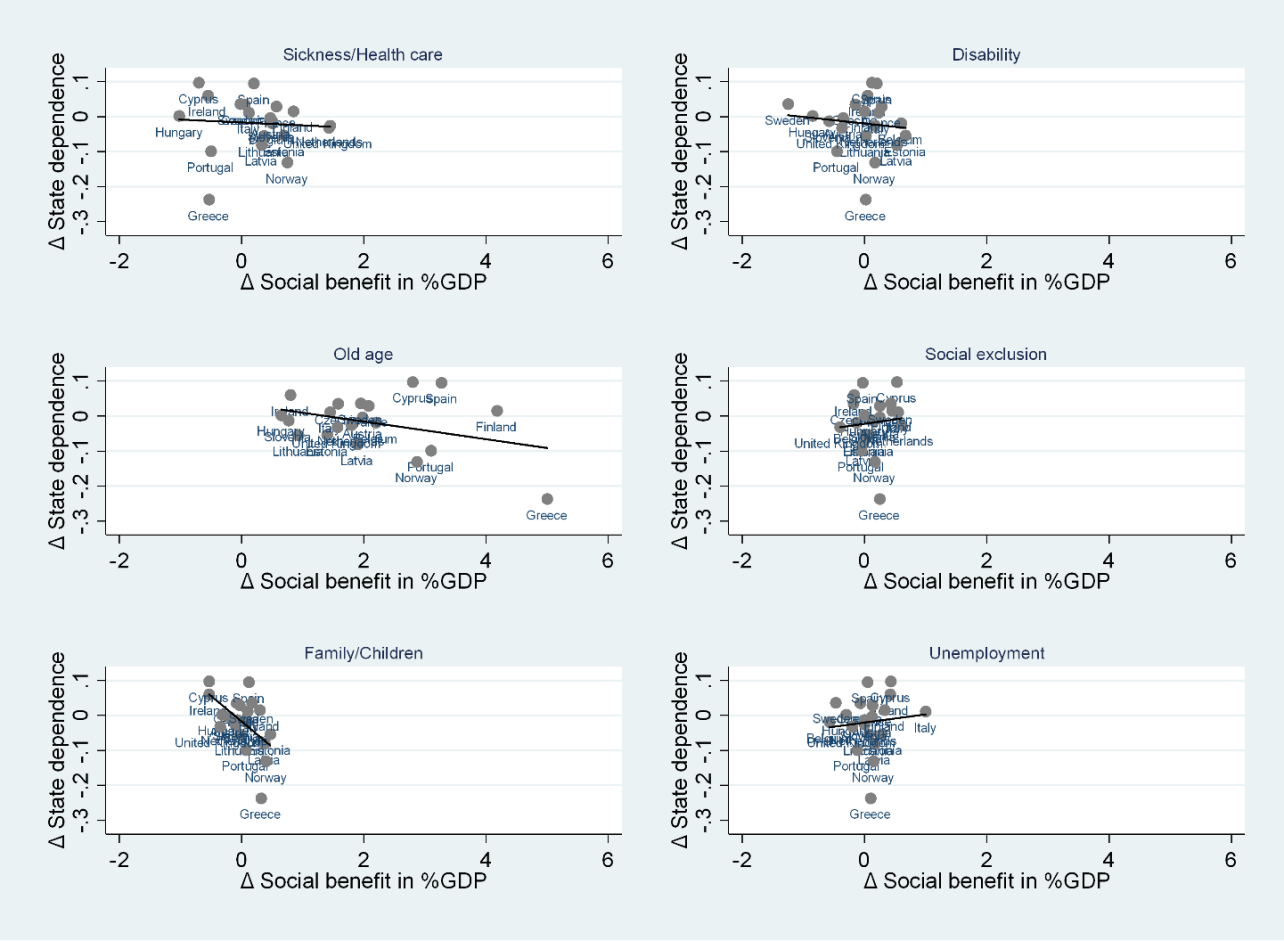
The correlation between state dependence variation and functions of social benefit variation. Source: Authors’ calculations from EU-SILC 2005–2008 and EU-SILC 2015–2018 data

Our results are shown in Fig. 3, as well as in Figs. 4 and 5. Firstly, we report the relationships between the change in country-level genuine state dependence and the change in social benefits expressed as a percentage of GDP, change in GDP growth, and change in the share of temporary employment, respectively. According to Fig. 3, we see that variation in genuine state dependence is negatively associated with variation in social benefits, while the correlation is positive with respect to the variation in GDP growth and weakly positive with respect to the share of temporary employment. This suggests that the support for disadvantaged groups was quite effective in lifting individuals out of poverty, while the advantages of economic growth were probably unequal along the income distribution and did not work to reduce genuine state dependence in the investigated period. Accordingly, Michálek and Výbošok (2019) found that in the investigated period, economic growth was accompanied by an increase in poverty in half of the EU member states.

We test the robustness of the mentioned associations to the presence of outliers by proceeding with the exclusion of one country at time. We find that the associations presented above are essentially confirmed.^11^ We also provide additional focus on the role of social benefits by analyzing the association between variation in genuine state dependence and variation in specific types of social benefits (Fig. 4), that is, means tested and non-means-tested ones on the one hand and cash and in-kind transfers on the other. We find the existence of a negative association between change in state dependence and variation in non-means-tested and cash social benefits, while the association with means-tested and in-kind social benefits is weakly positive. The signs of the mentioned associations generally hold, except for in-kind social benefits. In this respect, some studies (e.g., Fabrizi et al. 2014) have shown that means-tested measures are more effective to fight poverty and inequality for targeted categories, even though extensive means testing is not an essential condition for well-targeted transfers.

Finally, Fig. 5 reports the associations between variation in genuine state dependence and variation in social benefits identified according to their function (sickness/healthcare, disability, old age, social exclusion, family/children, and unemployment). Our graphs suggest that there are negative but weak correlations between variation in genuine state dependence and variation in social benefits for sickness/healthcare and disability, and weakly positive correlations for social exclusion and unemployment. In addition, the negative correlation is stronger when looking at social benefits for old age, but that relationship does not hold up to the exclusion of Greece. Finally, we find a quite strong and robust negative correlation between variation in genuine state dependence and variation in social benefits for family/children, suggesting the efficacy of supporting specific disadvantaged groups. The literature has emphasized that especially in Southern Europe, sickness, disability, unemployment, and household-related benefits have a relatively low impact on poverty (Heady et al. 2001) because they are usually undersized. However, means-tested family allowances could be one of the most effective measures for alleviating poverty if the number of recipients and the size of allowances are increased (Fabrizi et al. 2014).

## Conclusions

This paper provides novel evidence on whether and how the determinants of poverty persistence have changed in Europe between pre- and post-Great Recession periods. We apply alternative dynamic probit models accounting for endogenous initial conditions and correlated random effects to 2005–2008 and 2015–2018 EU-SILC longitudinal datasets in order to estimate genuine state dependence and uncover the role of observable and unobservable factors in the risk of poverty. Our estimates indicate that genuine state dependence has slightly increased between pre- and post-Great recession periods in Europe. The point estimates are a little different across methods, but the essence of the results is fully robust. Our analysis also shows that the scarring effect of poverty has increased over time.

On the one hand, these results confirm that policy measures able to lift individuals and households above the poverty line would be even more important for avoiding future economic difficulties because of the potential effect in the reduction of poverty persistence. On the other hand, they highlight that despite the intentions of the Europe 2020 Strategy, anti-poverty programs did not effectively fight the vicious cycle in which poverty itself may determine future poverty. This may be due to several factors, including undersized support measures, the difficulties of reemployment because of human capital depreciation—which followed the increase in long-term unemployment during the crisis—and the diffusion of low-paying jobs.

The analysis at the country level shows that the increase in genuine state dependence at the European level is the result of a composite pattern. Among the twenty EU countries we analyze, genuine state dependence increased in some and decreased in others. However, the tendency for some countries to have strong genuine state dependence and for others to have relatively low genuine state dependence also emerged. Cross-country variability in genuine state dependence has decreased in the post-Great Recession period, suggesting that its relevance for poverty persistence has become more homogeneous across countries over time. We do not find a consistent pattern of genuine state dependence within macro-regions, either in terms of magnitude or evolution. The evolution of genuine state dependence, however, appears to be correlated with some macro-variables. In particular, a decrease in genuine state dependence is associated with an increase in social benefits (expressed in % GDP), while the acceleration of GDP growth appears to be correlated with an increase in genuine state dependence, suggesting that the benefits of economic growth were unequal along the income distribution in the post-Great Recession period. The contribution of social benefits to the reduction of genuine state dependence was not homogeneous, but it quite clearly emerges that cash transfers and support for families and children appear to be more effective. Obviously, we are aware that we cannot offer a causal interpretation for these relationships, but they may provide new insights to be better explored in future works.

Policies aimed at fighting poverty should enhance individual and household characteristics that are protective against poverty. Our results confirm the protective roles of higher education, employment stability, and childcare, among other factors. However, some remarkable changes can be noted between before and after the Great Recession. It is important to stress that the contrasting role of higher education has diminished during the post-Great Recession period, possibly as a consequence of a reduction in returns to education. This highlights the relevance of monitoring the effectiveness of higher education in avoiding unemployment and low-paying jobs and calls into question both the adequacy of educational systems and the functioning of labor markets, as well as the efficacy of related policies. In contrast, the protective role of stable employment has increased slightly, indicating that labor market segmentation continues to represent an issue in its interaction with poverty-related policies. Finally, we find that the presence of children increases the risk of poverty. However, it emerged that the impact has almost doubled over time for children aged 0–3, pointing to the importance of supporting families with young children in order to combat poverty. The reduction of child poverty appears even more important when considering its effects on cognitive and non-cognitive skill formation and the possible long-term consequences along an individual’s life.

## Footnotes

1 Figures available online at https://ec.europa.eu/eurostat/databrowser/view/ilc_li02/default/table?lang=en.

2 See Jenkins and Van Kerm (2014) for a discussion of alternative EU indicators of persistent and current poverty.

3 This is a standard equivalence scale to calculate the number of “equivalent adults” in a household. Such a scale assigns a weight of 1.0 to the first adult, 0.5 to the second and each subsequent person aged 14 and over, and 0.3 to each child under 14.

4 Bryan and Jenkins (2016) show that in the presence of many individuals but a relatively low number of countries (less than 30), the robustness of country effects may be questioned in multilevel modelling, while the estimation of individual-level effects is reliable. Other sources cite rules of thumb that recommend the use of between 10 and 50 groups as a minimum (e.g., Hox 2010).

5 Albarran et al. (2019) proposed an alternative method to handle potential bias in a dynamic probit random effects model with unbalanced panels. Their method produces consistent estimates by allowing the process that determines the unbalanced structure of the data to be correlated with the permanent unobserved heterogeneity. We use this method as a robustness check, as mentioned in Sect. 5.

6 We run Z tests in all of our comparisons by following the formula: $Z={(\beta }_{1}-{\beta }_{2})/\sqrt{{\left(SE{\beta }_{1}\right)}^{2}+{\left(SE{\beta }_{2}\right)}^{2}}$ (Clogg et al., 1995). In Table 3, we report the significance levels of the differences between the two independent samples. The two samples are disjointed as they come from two different EU-SILC four-year panels, i.e., 2005/2008 and 2015/2018.

7 As robustness checks, we test the significance of the changes in the post-recession period by regressing a full-interaction (Wooldridge) model, for which each covariate is interacted with a dummy variable taking a value of one if the observation is related to the post-recession period. We note an increase in genuine state dependence after the Great Recession that is statistically significant. For the sake of brevity, we do not report these results but they are available upon request.

8 From Table 3, we note that genuine state dependence increases from 6.8 pp. to 7.2 pp. according to the Rabe-Hesketh and Skrondal model, while it increases from 8 pp. to 8.7 pp. according to the hierarchical model.

9 As explained in Sect. 4, the Wooldridge model specification includes Mundlak’s correction for the average of time-varying covariates. For the sake of brevity, we do not report the AMEs for such variables but these must be considered when interpreting the results. For the number of disabled persons, the sign was negative in both periods.

10 The three indicators we consider are representative of different sources of income formation and their evolution across the pre- and post-Great Recession periods (e.g., Jenkins 2020).

11 Graphical tests are available upon request.
